# Successful treatment of primary mediastinal malignant germ cell tumor with multiple systemic metastases in children using multi-drug combination chemotherapy: a rare case report

**DOI:** 10.3389/fphar.2026.1776028

**Published:** 2026-03-25

**Authors:** Yang-Yang Jiao, Yan-Hua Li, Xue-Lian Liao, Jing-Bo Shao, Ting Zhang, Jing-Wei Yang, Can Huang, Wen-Yan Huang, Sha-Yi Jiang

**Affiliations:** 1 Department of Hematology and Oncology, Shanghai Children’s Hospital, School of medicine, Shanghai Jiao Tong University, Shanghai, China; 2 Department of Nephrology, Rheumatology and Immunology, Shanghai Children’s Hospital, School of Medicine, Shanghai Jiao Tong University, Shanghai, China

**Keywords:** case report, children, malignant germ cell tumor, mediastinal, treatment

## Abstract

Primary mediastinal malignant germ cell tumors (PMMGCTs) in children are highly aggressive and associated with a poor prognosis. We herein report the case of a male pediatric patient who presented with a large mediastinal mass and extensive metastases to the lungs, brain, kidneys, and multiple bones, illustrating the aggressive nature of this disease. The patient received a multi-drug combination chemotherapy regimen before and after tumor resection and successfully achieved complete remission. Notably, he has survived for 12 months and remains in complete remission to date. This case underscores the potential efficacy of multi-agent combination chemotherapy as a component of a multimodal treatment strategy for advanced PMMGCTs.

## 1 Introduction

Primary mediastinal malignant germ cell tumors (PMMGCTs), which are thought to originate from primordial germ cells that aberrantly migrate during embryogenesis, represent a distinct and clinically significant entity among extragonadal germ cell tumors (GCTs) (Bhuta et al., 2023). They constitute approximately 3%–6% of all extragonadal GCTs (De Pasquale et al., 2016; Depani et al., 2019; Faure-Conter et al., 2023; Jiang et al., 2023; Dao et al., 2025) and are notably one of the most prevalent mediastinal tumors in the pediatric population, accounting for up to 27.6% of mediastinal tumors (Jiang et al., 2024). While PMMGCTs are rare in adolescents, the mediastinum persists as the most common site for extragonadal malignant germ cell tumors (MGCTs) within this age group (Zhao et al., 2023). A critical clinical challenge stems from the pronounced disparity between the generally favorable prognosis of pediatric MGCTs overall and the well-established fact that a primary mediastinal site is an independent predictor of poor outcome. This contradiction highlights the substantial and persistent challenges PMMGCTs present to multidisciplinary care teams, underscoring the need for tailored and intensive therapeutic approaches. In this article, we present a case of extensively metastatic PMMGCT successfully treated with multimodal therapy, including multi-drug combination chemotherapy, offering a reference for clinicians.

## 2 Case description

**FIGURE 1:**
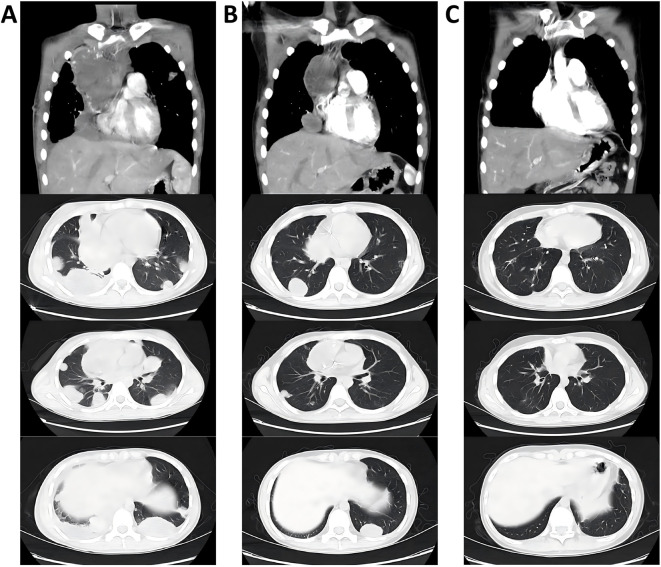
Dynamic CT evaluation of mediastinal mass and pulmonary lesions. **(A)** at diagnosis. **(B)** after six cycles of NACT. **(C)** five months after the end of treatment.

**FIGURE 2:**
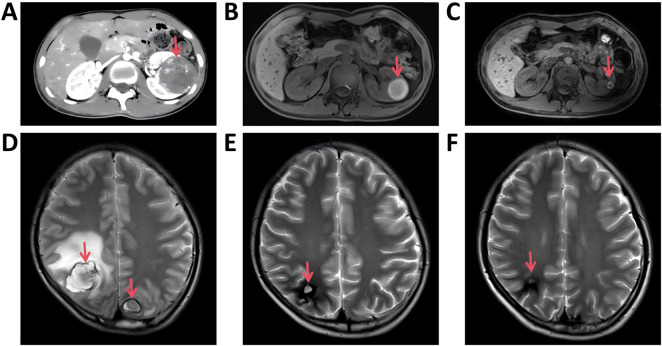
Dynamic imaging evaluation of renal lesions and cerebral lesions. **(A,D)** at diagnosis. **(B,E)** after six cycles of NACT. **(C,F)** five months after the end of treatment.

**FIGURE 3:**
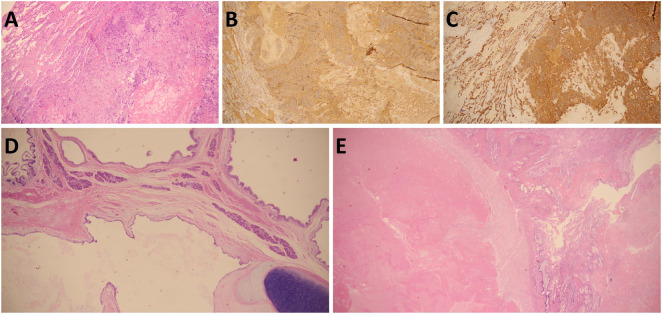
Pathological findings at diagnosis **(A–C)** and after six cycles of NACT **(D,E)**. **(A)** H&E staining of the lung lesion revealed features consistent with choriocarcinoma. **(B)** IHC staining showed positivity for HCG. **(C)** IHC staining showed positivity for cytokeratin. **(D)** H&E staining of the mediastinal mass revealed a residual componet of 10% mature teratoma. **(E)** H&E staining of the pulmonary metastases showed extensive necrosis.

A 15-year-old male patient presented with the complaint of back pain. Physical examination revealed mild tenderness on palpation over the back. Serum tumor markers analysis revealed markedly elevated β-human chorionic gonadotropin (β-hCG) (>10000MIU/mL) and moderately elevated alpha-fetoprotein (AFP) (33 ng/mL). Analysis of cerebrospinal fluid (CSF) revealed elevated β-hCG (>1,345 mIU/mL) and normal AFP (<0.5 ng/mL). Chest computed tomography (CT) scan (Figure 1A) demonstrated an expansive mass (89 × 56 × 104 mm) in the right anterior mediastinum, accompanied by multiple pulmonary metastatic masses. Abdominal CT scan (Figure 2A) confirmed multiple masses and nodules in both kidneys. Cranial magnetic resonance imaging (MRI) (Figure 2D) revealed lesions in the bilateral parietal lobes and abnormal signal intensities in the left occipital lobe and left cerebellar hemisphere, with the largest lesion measuring 25.6 × 29.3 × 37.1 mm. Positron emission tomography-computed tomography (PET-CT) demonstrated high uptake of 18F-fluorodeoxyglucose in an expansive mass of the right anterior mediastinum (maximum standardized uptake value [SUVmax] 5.8), as well as in bilateral pulmonary lesions, bilateral renal masses, and multiple osseous sites including the L1 and L5 vertebrae and the right iliac crest. The nodule in the upper lobe of the right lung was resected via video-assisted thoracoscopic surgery (VATS), and histopathological examination confirmed the diagnosis of choriocarcinoma (Figures 3A–C). Given the presence of multiple distant metastases at diagnosis, the disease was staged as IV and the patient was stratified into the high-risk prognostic group.

The patient completed a total of nine cycles of multi-agent chemotherapy, with alternating BEP, AVCP, and IVE protocols administered every 21 days. The BEP regimen consisted of cisplatin (20 mg/m^2^/day, days 1–5), etoposide (100 mg/m^2^/day, days 1–5), and bleomycin (15 mg/m^2^/day, days 1, 8, and 15). The AVCP regimen included doxorubicin (30 mg/m^2^/day, days 1 and 8), vincristine (1.5 mg/m^2^/day, days 0 and 7), cyclophosphamide (300 mg/m^2^/day, days 1–3), and cisplatin (90 mg/m^2^/day, day 0). The IVE regimen comprised ifosfamide (1.5 g/m^2^/day, days 1–5), vincristine (1.5 mg/m^2^/day, days 0 and 7), and etoposide (100 mg/m^2^/day, days 1–5). A prophylactic intrathecal injection of methotrexate was administered via lumbar puncture during each chemotherapy cycle. Throughout the treatment period, no non-hematological grade 3 or 4 adverse events were observed.

**FIGURE 4:**
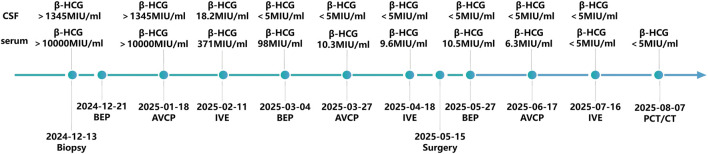
Timeline of the treatment and β-hCG trends in this case.

Following six cycles of neoadjuvant chemotherapy (NACT), chest CT scan revealed a reduction in the size of the mediastinal mass (measuring 71.9 × 43.7 × 59.3 mm) along with a decrease in both the number and dimensions of pulmonary metastatic foci (Figure 1B). Meanwhile, MRI imaging demonstrated shrinkage of the metastatic lesions in both kidneys (Figure 2B) and the brain (Figure 2E). The patient subsequently underwent tumor resection via VATS to remove the mediastinal mass and multiple lung metastases. Given the significant chemotherapy-induced tumor shrinkage, the residual renal and intracranial metastases were managed non-surgically. Histopathological examination of the resected mediastinal tumor revealed a residual mature teratoma component (approximately 10%) (Figure 3D). Similarly, the pulmonary metastases exhibited extensive necrosis (Figure 3F), with no viable malignant cells identified in any of the examined samples. Postoperatively, the patient completed three additional cycles of chemotherapy. Follow-up PET/CT scans confirmed complete remission (CR). The treatment timeline and β-hCG trends are summarized in Figure 4.

The patient has been under regular surveillance with serial serum biomarker assessments and imaging studies. At the most recent follow-up, 5 months after the end of treatment, there is no evidence of disease recurrence or progression (Figure 1C, Figures 2C, F).

## 3 Discussion

The introduction of cisplatin-based chemotherapy in the 1970s represented a pivotal advancement in the management of MGCTs, with contemporary overall survival (OS) rates now exceeding 90% (Dao et al., 2025). Despite this substantial improvement in overall outcomes, PMMGCTs continue to be associated with a significantly worse prognosis (Grabski et al., 2017; Huang et al., 2022). Data from three major pediatric centers in Shanghai further confirm that both event-free survival (EFS) and OS are markedly inferior for patients with mediastinal MGCTs compared to those with non-mediastinal primary tumors (Jiang et al., 2023). PMMGCTs are histologically categorized into seminomas and non-seminomatous germ cell tumors (NSGCTs). NSGCTs, which encompass yolk sac tumors, choriocarcinoma, embryonal carcinoma, and mixed malignant germ cell tumors, are typically associated with a less favorable prognosis than seminomas. Population-based analyses from the Surveillance, Epidemiology, and End Results (SEER) registry corroborate the poor survival outcomes of mediastinal NSGCTs, with reported 3-year and 5-year OS rates of 63.1% and 61.2%, respectively. A stark contrast is observed between histological subtypes: mediastinal seminoma exhibits a 5-year OS of 100%, whereas 5-year OS for NSGCTs ranges from 20.5% to 61.6% (Wu et al., 2023). In alignment with these trends, data from a Chinese cohort similarly indicated a 100% OS rate for mediastinal seminoma, while patients with NSGCTs achieved OS and disease-free survival (DFS) rates of 74.2% and 64.7%, respectively (Huang et al., 2017).

Research on metastatic MGCTs indicates that the metastasis rate of PMMGCTs is significantly higher than that of MGCTs originating from other primary sites. Among patients aged ≥8 years, the metastasis rate of mediastinal MGCTs is notably elevated, reaching 38.8%. Specifically, distant metastasis rates vary across histological subtypes but are generally elevated in mediastinal MGCTs, particularly for mediastinal choriocarcinoma (84.4%) and mediastinal yolk sac tumors (42.9%).

Mediastinal choriocarcinoma demonstrates a particularly pronounced propensity for brain metastasis, with an organ tropism metastasis rate as high as 56% (Park, 2025). This patient exhibited several high-risk features characteristic of aggressive malignant germ cell tumors: a primary mediastinal location, non-seminomatous pathology with choriocarcinoma components, a markedly elevated serum β-hCG level, and widespread metastatic disease at initial presentation. Collectively, these factors constituted a “perfect storm” of poor prognostic indicators, historically associated with dismal outcomes. The presence of choriocarcinoma was particularly concerning due to its well-documented association with highly aggressive biological behavior, propensity for hematogenous dissemination, and poor treatment response (Tseng et al., 2025), especially its proclivity for spreading to the central nervous system (CNS). The confluence of these factors placed this patient into the highest-risk category.

The treatment of poor-prognosis extracranial MGCTs remains challenging, prompting extensive clinical exploration internationally. Investigations including high-dose cisplatin regimens and the addition of cyclophosphamide to PEB regimens have not demonstrated a survival benefit for high-risk MGCTs, including PMMGCTs (Malogolowkin et al., 2013; Prior et al., 2025). Adolescent patients with metastatic GCTs exhibit biological and clinical characteristics more akin to young adults than to children, leading to recommendations that adolescent males with GCTs should be managed according to adult treatment protocols (Shaikh et al., 2021). Building upon the classic BEP regimen established for adults and drawing from previous treatment strategies employed by two specialized centers in Shanghai (Jiang et al., 2023), we explored a multi-drug combination therapy for pediatric mediastinal MGCTs.

The therapeutic strategy employed in this case involved the rotational use of BEP, AVCP, and IVE regimens. The rationale for this approach was the significant tumor heterogeneity typically present in PMMGCT, where distinct cellular components exhibited differential chemosensitivity. By utilizing regimens with non-overlapping mechanisms of action, the strategy aimed to deliver multi-targeted cytotoxicity, potentially mitigating the risk of intrinsic or acquired drug resistance. Furthermore, this rotational design maintained a high overall dose intensity while distributing the cumulative toxicities specific to individual agents—particularly the pulmonary toxicity associated with bleomycin and the nephrotoxicity and neurotoxicity linked to cisplatin—thereby potentially improving treatment tolerability. The rapid decline in serum β-hCG levels, significant tumor reduction on imaging, and postoperative pathological findings confirming mature teratoma and necrotic tissue in the resected specimen collectively provide compelling evidence for the efficacy of this chemotherapeutic strategy.

The probability of intracranial metastasis at the initial diagnosis of MGCTs is approximately 1%. For patients with asymptomatic brain metastases identified at initial presentation, systemic chemotherapy alone is typically employed as the first-line treatment. Subsequent interventions, such as surgical resection or radiotherapy, are usually reserved for cases where complete remission is not achieved with chemotherapy alone (Le et al., 2022; Salous and Adra, 2024). In this pediatric case, we adopted a combined therapeutic approach consisting of systemic chemotherapy alongside intrathecal methotrexate administration via lumbar puncture. This strategy was informed by treatment protocols for cerebral metastases from gestational trophoblastic neoplasia in adults (Xiao et al., 2022). The treatment response was monitored by tracking trends in CSF β-hCG levels, which normalized after three treatment cycles and remained normal thereafter.

Finally, while the 12-month survival outcome is highly encouraging, patients with PMMGCT remain at risk for long-term recurrence, necessitating extended follow-up. Furthermore, although no non-hematological grade 3 or 4 toxicities occurred during treatment, the potential for therapy-related toxicities warrants careful monitoring in long-term follow-up. Furthermore, the findings and success of this therapeutic approach, while promising, are derived from a single case report and thus have inherent limitations in generalizability. Their efficacy and safety profile require validation in larger prospective studies.

## 4 Conclusion

In summary, this case demonstrates that primary mediastinal malignant germ cell tumor with extensive metastases, despite its historically poor prognosis, can be curatively managed through an integrated, multimodal therapeutic approach comprising conventional-dose, multi-agent alternating chemotherapy, timely surgical intervention, and aggressive central nervous system prophylaxis. This successful outcome provides hope and a valuable treatment paradigm for managing this challenging disease entity.

## Data Availability

The original contributions presented in the study are included in the article/supplementary material, further inquiries can be directed to the corresponding authors.
